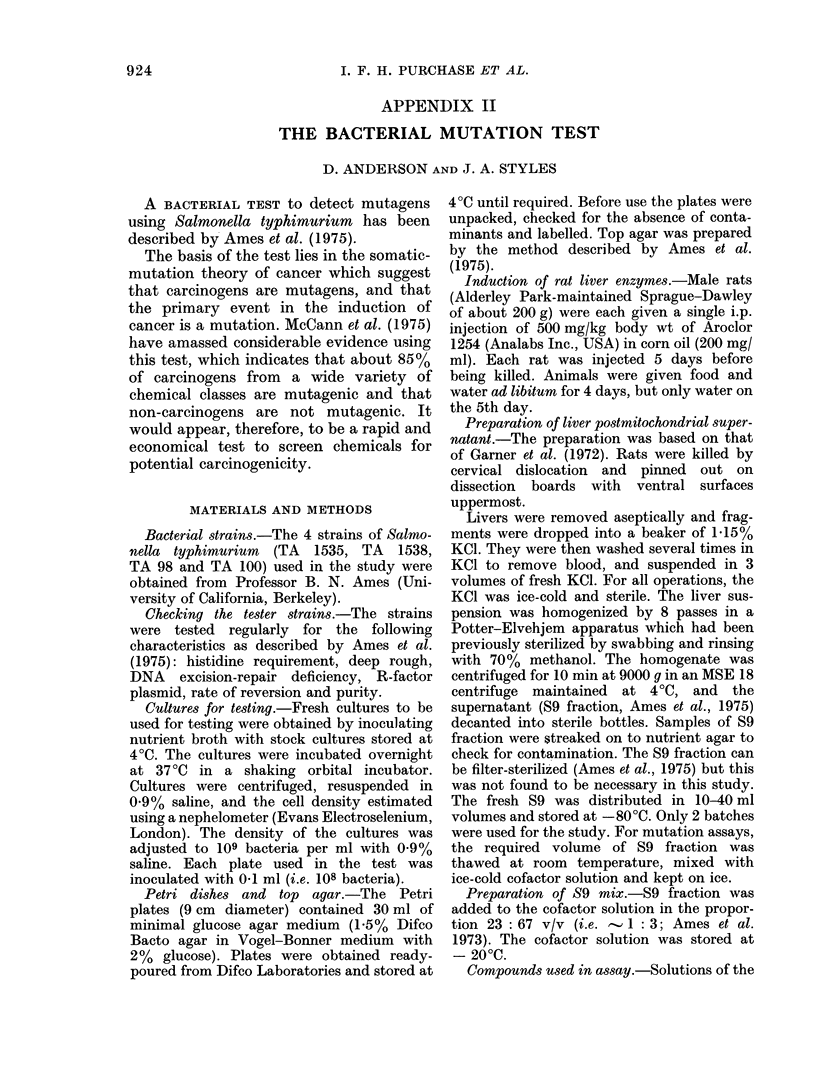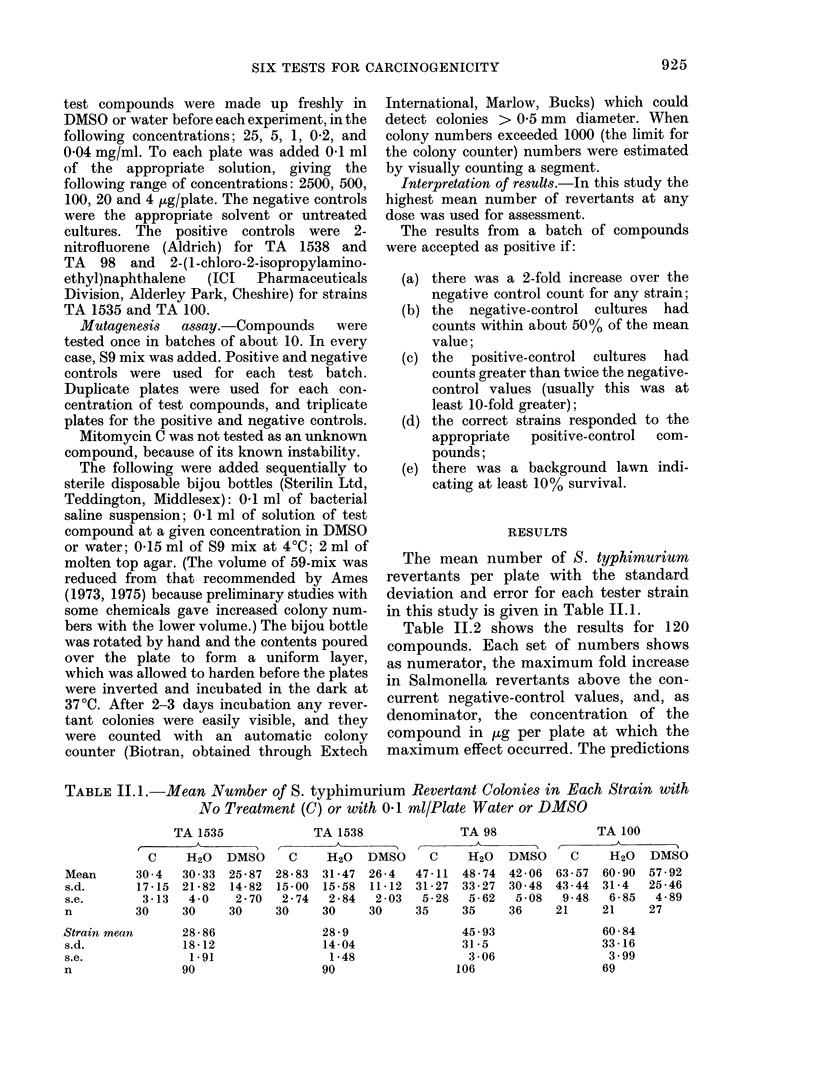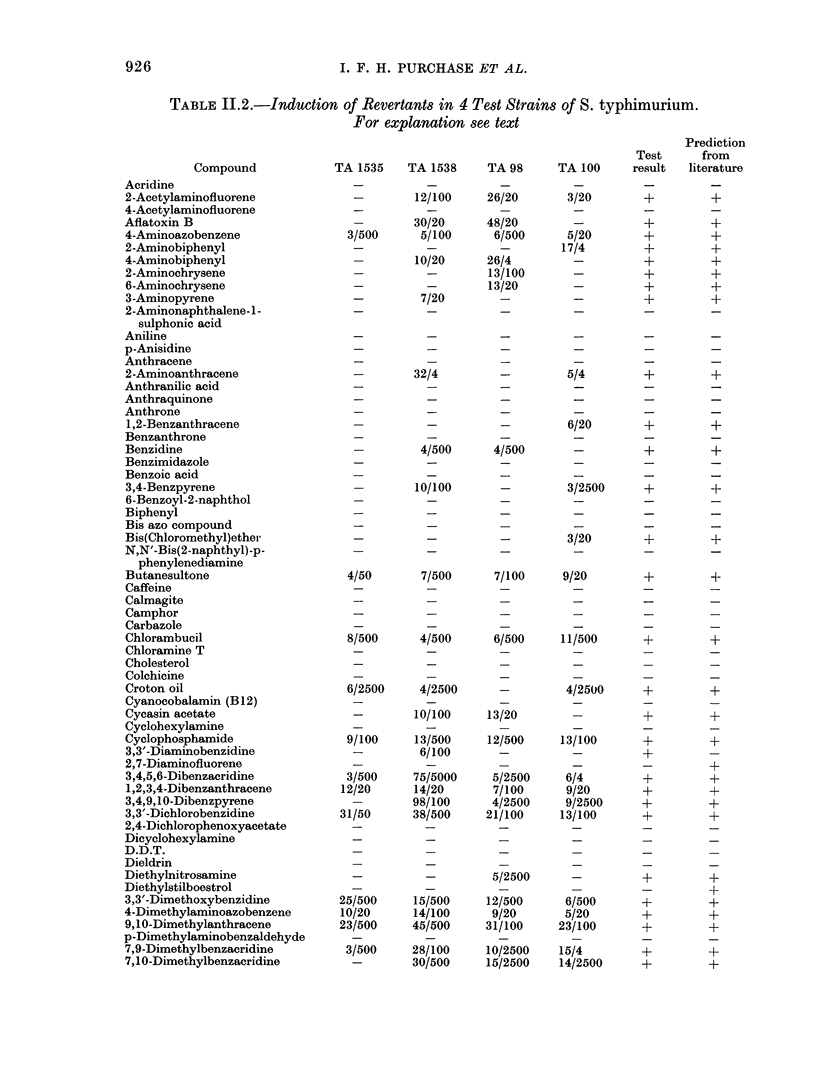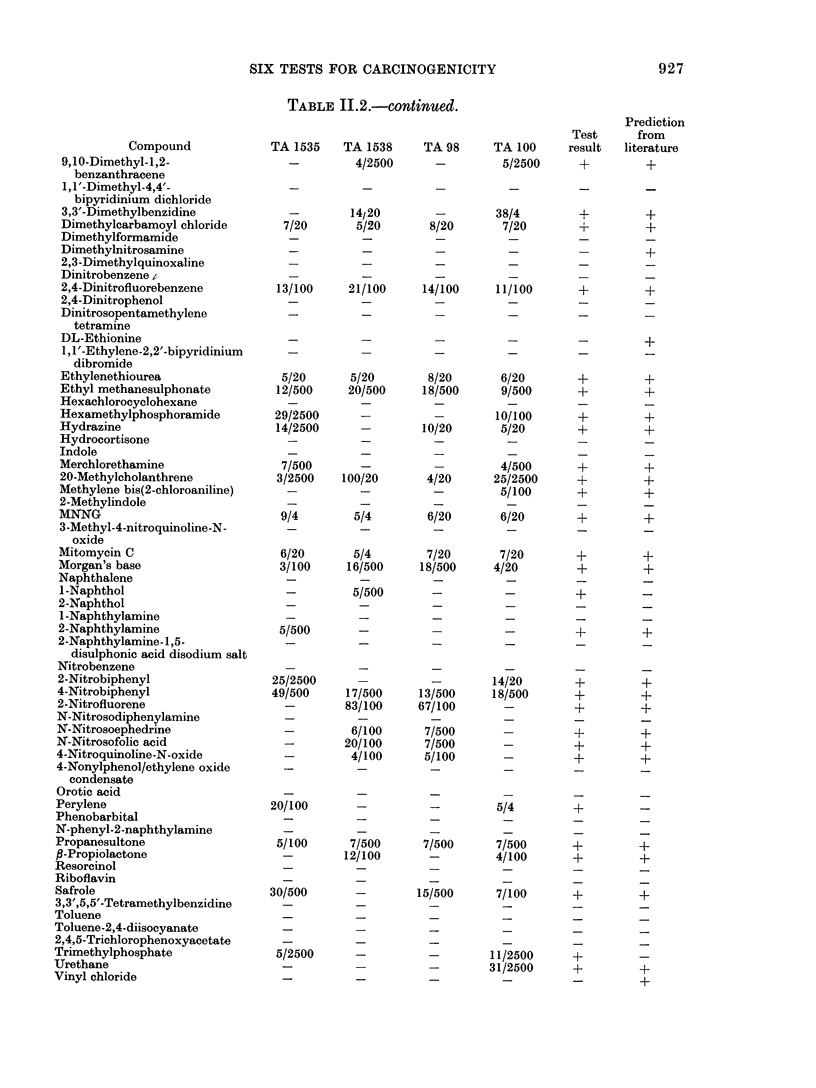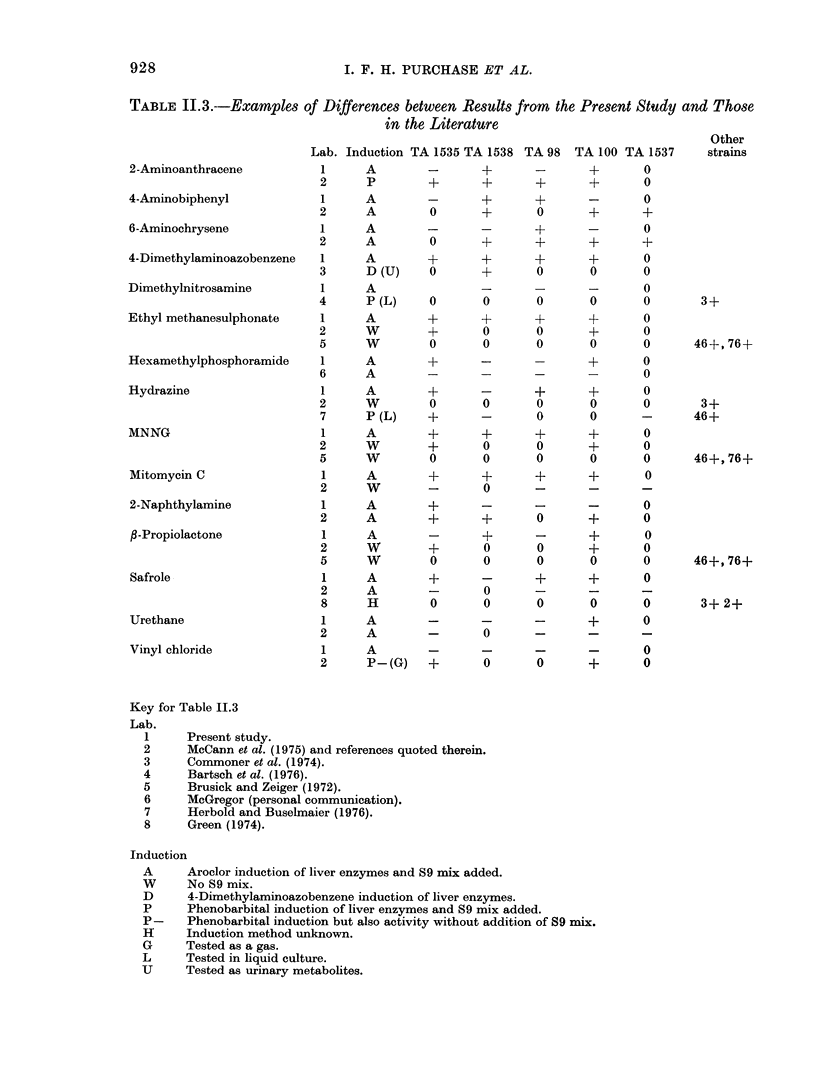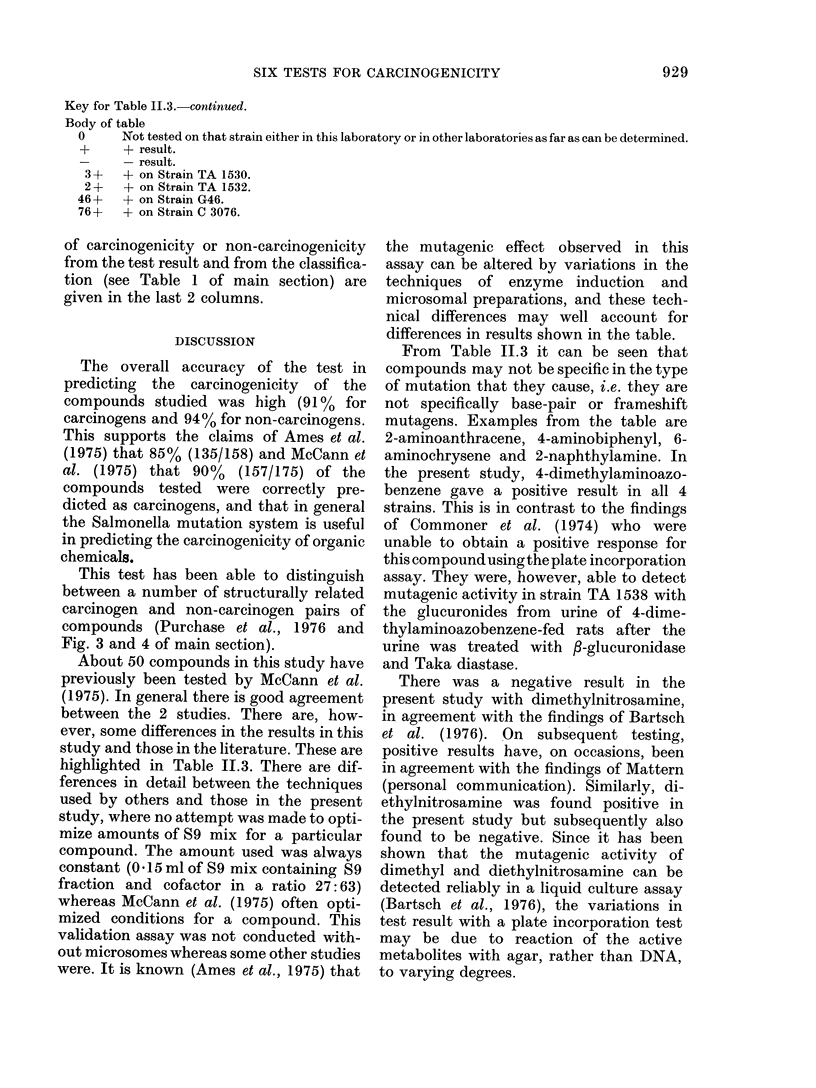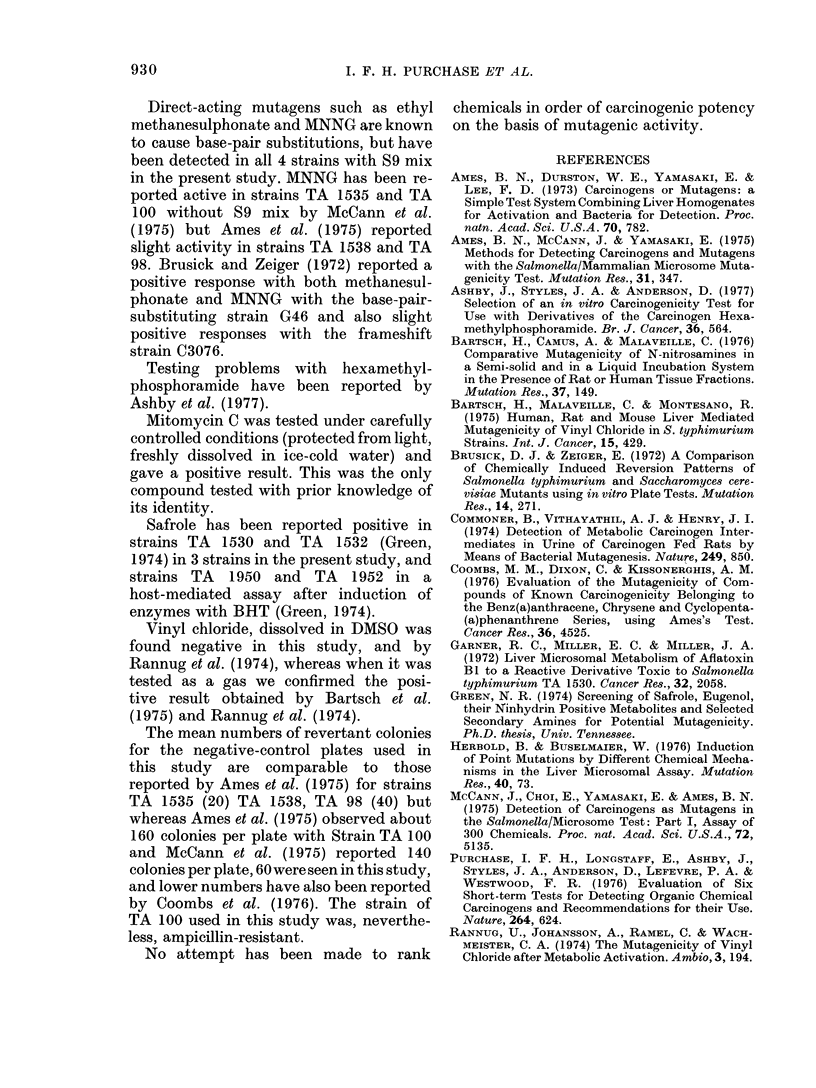# The bacterial mutation test. Six tests for carcinogenicity.

**DOI:** 10.1038/bjc.1978.134

**Published:** 1978-06

**Authors:** D. Anderson, J. A. Styles


### APPENDIX II

### THE BACTERIAL MUTATION TEST

D. ANDERSON AND J. A. STYLES

A BACTERIAL TEST to detect mutagens using *Salmonella typhimurium* has been described by Ames *et al.* (1975).

The basis of the test lies in the somatic-mutation theory of cancer which suggest that carcinogens are mutagens, and that the primary event in the induction of cancer is a mutation. McCann *et al.* (1975) have amassed considerable evidence using this test, which indicates that about 85% of carcinogens from a wide variety of chemical classes are mutagenic and that non-carcinogens are not mutagenic. It would appear, therefore, to be a rapid and economical test to screen chemicals for potential carcinogenicity.

### MATERIALS AND METHODS

*Bacterial strains.*—The 4 strains of *Salmonella typhimurium* (TA 1535, TA 1538, TA 98 and TA 100) used in the study were obtained from Professor B. N. Ames (University of California, Berkeley).

*Checking the tester strains.*—The strains were tested regularly for the following characteristics as described by Ames *et al.* (1975): histidine requirement, deep rough, DNA excision-repair deficiency, R-factor plasmid, rate of reversion and purity.

*Cultures for testing.*—Fresh cultures to be used for testing were obtained by inoculating nutrient broth with stock cultures stored at 4°C. The cultures were incubated overnight at 37°C in a shaking orbital incubator. Cultures were centrifuged, resuspended in 0·9% saline, and the cell density estimated using a nephelometer (Evans Electroselenium, London). The density of the cultures was adjusted to $10^9$ bacteria per ml with 0·9% saline. Each plate used in the test was inoculated with 0·1 ml (*i.e.* $10^8$ bacteria).

*Petri dishes and top agar.*—The Petri plates (9 cm diameter) contained 30 ml of minimal glucose agar medium (1·5% Difco Bacto agar in Vogel–Bonner medium with 2% glucose). Plates were obtained ready-poured from Difco Laboratories and stored at 4°C until required. Before use the plates were unpacked, checked for the absence of contaminants and labelled. Top agar was prepared by the method described by Ames *et al.* (1975).

*Induction of rat liver enzymes.*—Male rats (Alderley Park-maintained Sprague–Dawley of about 200 g) were each given a single i.p. injection of 500 mg/kg body wt of Aroclor 1254 (Analabs Inc., USA) in corn oil (200 mg/ml). Each rat was injected 5 days before being killed. Animals were given food and water *ad libitum* for 4 days, but only water on the 5th day.

*Preparation of liver postmitochondrial supernatant.*—The preparation was based on that of Garner *et al.* (1972). Rats were killed by cervical dislocation and pinned out on dissection boards with ventral surfaces uppermost.

Livers were removed aseptically and fragments were dropped into a beaker of 1·15% KCl. They were then washed several times in KCl to remove blood, and suspended in 3 volumes of fresh KCl. For all operations, the KCl was ice-cold and sterile. The liver suspension was homogenized by 8 passes in a Potter–Elvehjem apparatus which had been previously sterilized by swabbing and rinsing with 70% methanol. The homogenate was centrifuged for 10 min at 9000 *g* in an MSE 18 centrifuge maintained at 4°C, and the supernatant (S9 fraction, Ames *et al.*, 1975) decanted into sterile bottles. Samples of S9 fraction were streaked on to nutrient agar to check for contamination. The S9 fraction can be filter-sterilized (Ames *et al.*, 1975) but this was not found to be necessary in this study. The fresh S9 was distributed in 10–40 ml volumes and stored at −80°C. Only 2 batches were used for the study. For mutation assays, the required volume of S9 fraction was thawed at room temperature, mixed with ice-cold cofactor solution and kept on ice.

*Preparation of S9 mix.*—S9 fraction was added to the cofactor solution in the proportion 23 : 67 v/v (*i.e.* ~1 : 3; Ames *et al.* 1973). The cofactor solution was stored at − 20°C.

*Compounds used in assay.*—Solutions of the test compounds were made up freshly in DMSO or water before each experiment, in the following concentrations; 25, 5, 1, 0·2, and 0·04 mg/ml. To each plate was added 0·1 ml of the appropriate solution, giving the following range of concentrations: 2500, 500, 100, 20 and 4 μg/plate. The negative controls were the appropriate solvent or untreated cultures. The positive controls were 2-nitrofluorene (Aldrich) for TA 1538 and TA 98 and 2-(1-chloro-2-isopropylamino-ethyl)naphthalene (ICI Pharmaceuticals Division, Alderley Park, Cheshire) for strains TA 1535 and TA 100.

*Mutagenesis assay.*—Compounds were tested once in batches of about 10. In every case, S9 mix was added. Positive and negative controls were used for each test batch. Duplicate plates were used for each concentration of test compounds, and triplicate plates for the positive and negative controls.

Mitomycin C was not tested as an unknown compound, because of its known instability.

The following were added sequentially to sterile disposable bijou bottles (Sterilin Ltd, Teddington, Middlesex): 0·1 ml of bacterial saline suspension; 0·1 ml of solution of test compound at a given concentration in DMSO or water; 0·15 ml of S9 mix at 4°C; 2 ml of molten top agar. (The volume of 59-mix was reduced from that recommended by Ames (1973, 1975) because preliminary studies with some chemicals gave increased colony numbers with the lower volume.) The bijou bottle was rotated by hand and the contents poured over the plate to form a uniform layer, which was allowed to harden before the plates were inverted and incubated in the dark at 37°C. After 2–3 days incubation any revertant colonies were easily visible, and they were counted with an automatic colony counter (Biotran, obtained through Extech International, Marlow, Bucks) which could detect colonies > 0·5 mm diameter. When colony numbers exceeded 1000 (the limit for the colony counter) numbers were estimated by visually counting a segment.

*Interpretation of results.*—In this study the highest mean number of revertants at any dose was used for assessment.

The results from a batch of compounds were accepted as positive if:

(a) there was a 2-fold increase over the negative control count for any strain;
(b) the negative-control cultures had counts within about 50% of the mean value;
(c) the positive-control cultures had counts greater than twice the negative-control values (usually this was at least 10-fold greater);
(d) the correct strains responded to the appropriate positive-control compounds;
(e) there was a background lawn indicating at least 10% survival.

### RESULTS

The mean number of *S. typhimurium* revertants per plate with the standard deviation and error for each tester strain in this study is given in Table II.1.

Table II.2 shows the results for 120 compounds. Each set of numbers shows as numerator, the maximum fold increase in Salmonella revertants above the concurrent negative-control values, and, as denominator, the concentration of the compound in μg per plate at which the maximum effect occurred. The predictions

TABLE II.1.—*Mean Number of* S. typhimurium *Revertant Colonies in Each Strain with No Treatment (C) or with* 0·1 *ml/Plate Water or DMSO*

| | TA 1535 | | | TA 1538 | | | TA 98 | | | TA 100 | | |
|---|---|---|---|---|---|---|---|---|---|---|---|---|
| | C | $H_2O$ | DMSO | C | $H_2O$ | DMSO | C | $H_2O$ | DMSO | C | $H_2O$ | DMSO |
| Mean | 30·4 | 30·33 | 25·87 | 28·83 | 31·47 | 26·4 | 47·11 | 48·74 | 42·06 | 63·57 | 60·90 | 57·92 |
| s.d. | 17·15 | 21·82 | 14·82 | 15·00 | 15·58 | 11·12 | 31·27 | 33·27 | 30·48 | 43·44 | 31·4 | 25·46 |
| s.e. | 3·13 | 4·0 | 2·70 | 2·74 | 2·84 | 2·03 | 5·28 | 5·62 | 5·08 | 9·48 | 6·85 | 4·89 |
| n | 30 | 30 | 30 | 30 | 30 | 30 | 35 | 35 | 36 | 21 | 21 | 27 |
| *Strain mean* | | 28·86 | | | 28·9 | | | 45·93 | | | 60·84 | |
| s.d. | | 18·12 | | | 14·04 | | | 31·5 | | | 33·16 | |
| s.e. | | 1·91 | | | 1·48 | | | 3·06 | | | 3·99 | |
| n | | 90 | | | 90 | | | 106 | | | 69 | |

TABLE II.2.—*Induction of Revertants in 4 Test Strains of* S. typhimurium. *For explanation see text*

| Compound | TA 1535 | TA 1538 | TA 98 | TA 100 | Test result | Prediction from literature |
|---|---|---|---|---|---|---|
| Acridine | — | — | — | — | — | — |
| 2-Acetylaminofluorene | — | 12/100 | 26/20 | 3/20 | + | + |
| 4-Acetylaminofluorene | — | — | — | — | — | — |
| Aflatoxin B | — | 30/20 | 48/20 | — | + | + |
| 4-Aminoazobenzene | 3/500 | 5/100 | 6/500 | 5/20 | + | + |
| 2-Aminobiphenyl | — | — | — | 17/4 | + | + |
| 4-Aminobiphenyl | — | 10/20 | 26/4 | — | + | + |
| 2-Aminochrysene | — | — | 13/100 | — | + | + |
| 6-Aminochrysene | — | — | 13/20 | — | + | + |
| 3-Aminopyrene | — | 7/20 | — | — | + | + |
| 2-Aminonaphthalene-1-sulphonic acid | — | — | — | — | — | — |
| Aniline | — | — | — | — | — | — |
| p-Anisidine | — | — | — | — | — | — |
| Anthracene | — | — | — | — | — | — |
| 2-Aminoanthracene | — | 32/4 | — | 5/4 | + | + |
| Anthranilic acid | — | — | — | — | — | — |
| Anthraquinone | — | — | — | — | — | — |
| Anthrone | — | — | — | — | — | — |
| 1,2-Benzanthracene | — | — | — | 6/20 | + | + |
| Benzanthrone | — | — | — | — | — | — |
| Benzidine | — | 4/500 | 4/500 | — | + | + |
| Benzimidazole | — | — | — | — | — | — |
| Benzoic acid | — | — | — | — | — | — |
| 3,4-Benzpyrene | — | 10/100 | — | 3/2500 | + | + |
| 6-Benzoyl-2-naphthol | — | — | — | — | — | — |
| Biphenyl | — | — | — | — | — | — |
| Bis azo compound | — | — | — | — | — | — |
| Bis(Chloromethyl)ether | — | — | — | 3/20 | + | + |
| N,N′-Bis(2-naphthyl)-p-phenylenediamine | — | — | — | — | — | — |
| Butanesultone | 4/50 | 7/500 | 7/100 | 9/20 | + | + |
| Caffeine | — | — | — | — | — | — |
| Calmagite | — | — | — | — | — | — |
| Camphor | — | — | — | — | — | — |
| Carbazole | — | — | — | — | — | — |
| Chlorambucil | 8/500 | 4/500 | 6/500 | 11/500 | + | + |
| Chloramine T | — | — | — | — | — | — |
| Cholesterol | — | — | — | — | — | — |
| Colchicine | — | — | — | — | — | — |
| Croton oil | 6/2500 | 4/2500 | — | 4/2500 | + | + |
| Cyanocobalamin (B12) | — | — | — | — | — | — |
| Cycasin acetate | — | 10/100 | 13/20 | — | + | + |
| Cyclohexylamine | — | — | — | — | — | — |
| Cyclophosphamide | 9/100 | 13/500 | 12/500 | 13/100 | + | + |
| 3,3′-Diaminobenzidine | — | 6/100 | — | — | + | — |
| 2,7-Diaminofluorene | — | — | — | — | — | + |
| 3,4,5,6-Dibenzacridine | 3/500 | 75/5000 | 5/2500 | 6/4 | + | + |
| 1,2,3,4-Dibenzanthracene | 12/20 | 14/20 | 7/100 | 9/20 | + | + |
| 3,4,9,10-Dibenzpyrene | — | 98/100 | 4/2500 | 9/2500 | + | + |
| 3,3′-Dichlorobenzidine | 31/50 | 38/500 | 21/100 | 13/100 | + | + |
| 2,4-Dichlorophenoxyacetate | — | — | — | — | — | — |
| Dicyclohexylamine | — | — | — | — | — | — |
| D.D.T. | — | — | — | — | — | — |
| Dieldrin | — | — | — | — | — | — |
| Diethylnitrosamine | — | — | 5/2500 | — | + | + |
| Diethylstilboestrol | — | — | — | — | — | + |
| 3,3′-Dimethoxybenzidine | 25/500 | 15/500 | 12/500 | 6/500 | + | + |
| 4-Dimethylaminoazobenzene | 10/20 | 14/100 | 9/20 | 5/20 | + | + |
| 9,10-Dimethylanthracene | 23/500 | 45/500 | 31/100 | 23/100 | + | + |
| p-Dimethylaminobenzaldehyde | — | — | — | — | — | — |
| 7,9-Dimethylbenzacridine | 3/500 | 28/100 | 10/2500 | 15/4 | + | + |
| 7,10-Dimethylbenzacridine | — | 30/500 | 15/2500 | 14/2500 | + | + |

TABLE II.2.—*continued.*

| Compound | TA 1535 | TA 1538 | TA 98 | TA 100 | Test result | Prediction from literature |
|---|---|---|---|---|---|---|
| 9,10-Dimethyl-1,2-benzanthracene | — | 4/2500 | — | 5/2500 | + | + |
| 1,1′-Dimethyl-4,4′-bipyridinium dichloride | — | — | — | — | — | — |
| 3,3′-Dimethylbenzidine | — | 14/20 | — | 38/4 | + | + |
| Dimethylcarbamoyl chloride | 7/20 | 5/20 | 8/20 | 7/20 | + | + |
| Dimethylformamide | — | — | — | — | — | — |
| Dimethylnitrosamine | — | — | — | — | — | + |
| 2,3-Dimethylquinoxaline | — | — | — | — | — | — |
| Dinitrobenzene | — | — | — | — | — | — |
| 2,4-Dinitrofluorebenzene | 13/100 | 21/100 | 14/100 | 11/100 | + | + |
| 2,4-Dinitrophenol | — | — | — | — | — | — |
| Dinitrosopentamethylene tetramine | — | — | — | — | — | — |
| DL-Ethionine | — | — | — | — | — | + |
| 1,1′-Ethylene-2,2′-bipyridinium dibromide | — | — | — | — | — | — |
| Ethylenethiourea | 5/20 | 5/20 | 8/20 | 6/20 | + | + |
| Ethyl methanesulphonate | 12/500 | 20/500 | 18/500 | 9/500 | + | + |
| Hexachlorocyclohexane | — | — | — | — | — | — |
| Hexamethylphosphoramide | 29/2500 | — | — | 10/100 | + | + |
| Hydrazine | 14/2500 | — | 10/20 | 5/20 | + | + |
| Hydrocortisone | — | — | — | — | — | — |
| Indole | — | — | — | — | — | — |
| Merchlorethamine | 7/500 | — | — | 4/500 | + | + |
| 20-Methylcholanthrene | 3/2500 | 100/20 | 4/20 | 25/2500 | + | + |
| Methylene bis(2-chloroaniline) | — | — | — | 5/100 | + | + |
| 2-Methylindole | — | — | — | — | — | — |
| MNNG | 9/4 | 5/4 | 6/20 | 6/20 | + | + |
| 3-Methyl-4-nitroquinoline-N-oxide | — | — | — | — | — | — |
| Mitomycin C | 6/20 | 5/4 | 7/20 | 7/20 | + | + |
| Morgan's base | 3/100 | 16/500 | 18/500 | 4/20 | + | + |
| Naphthalene | — | — | — | — | — | — |
| 1-Naphthol | — | 5/500 | — | — | + | — |
| 2-Naphthol | — | — | — | — | — | — |
| 1-Naphthylamine | — | — | — | — | — | — |
| 2-Naphthylamine | 5/500 | — | — | — | + | + |
| 2-Naphthylamine-1,5-disulphonic acid disodium salt | — | — | — | — | — | — |
| Nitrobenzene | — | — | — | — | — | — |
| 2-Nitrobiphenyl | 25/2500 | — | — | 14/20 | + | + |
| 4-Nitrobiphenyl | 49/500 | 17/500 | 13/500 | 18/500 | + | + |
| 2-Nitrofluorene | — | 83/100 | 67/100 | — | + | + |
| N-Nitrosodiphenylamine | — | — | — | — | — | — |
| N-Nitrosoephedrine | — | 6/100 | 7/500 | — | + | + |
| N-Nitrosofolic acid | — | 20/100 | 7/500 | — | + | + |
| 4-Nitroquinoline-N-oxide | — | 4/100 | 5/100 | — | + | + |
| 4-Nonylphenol/ethylene oxide condensate | — | — | — | — | — | — |
| Orotic acid | — | — | — | — | — | — |
| Perylene | 20/100 | — | — | 5/4 | + | — |
| Phenobarbital | — | — | — | — | — | — |
| N-phenyl-2-naphthylamine | — | — | — | — | — | — |
| Propanesultone | 5/100 | 7/500 | 7/500 | 7/500 | + | + |
| β-Propiolactone | — | 12/100 | — | 4/100 | + | + |
| Resorcinol | — | — | — | — | — | — |
| Riboflavin | — | — | — | — | — | — |
| Safrole | 30/500 | — | 15/500 | 7/100 | + | + |
| 3,3′,5,5′-Tetramethylbenzidine | — | — | — | — | — | — |
| Toluene | — | — | — | — | — | — |
| Toluene-2,4-diisocyanate | — | — | — | — | — | — |
| 2,4,5-Trichlorophenoxyacetate | — | — | — | — | — | — |
| Trimethylphosphate | 5/2500 | — | — | 11/2500 | + | — |
| Urethane | — | — | — | 31/2500 | + | + |
| Vinyl chloride | — | — | — | — | — | + |

TABLE II.3.—*Examples of Differences between Results from the Present Study and Those in the Literature*

| | Lab. | Induction | TA 1535 | TA 1538 | TA 98 | TA 100 | TA 1537 | Other strains |
|---|---|---|---|---|---|---|---|---|
| 2-Aminoanthracene | 1 | A | — | + | — | + | 0 | |
| | 2 | P | + | + | + | + | 0 | |
| 4-Aminobiphenyl | 1 | A | — | + | + | — | 0 | |
| | 2 | A | 0 | + | 0 | + | + | |
| 6-Aminochrysene | 1 | A | — | — | + | — | 0 | |
| | 2 | A | 0 | + | + | + | + | |
| 4-Dimethylaminoazobenzene | 1 | A | + | + | + | + | 0 | |
| | 3 | D (U) | 0 | + | 0 | 0 | 0 | |
| Dimethylnitrosamine | 1 | A | | — | — | — | 0 | |
| | 4 | P (L) | 0 | 0 | 0 | 0 | 0 | 3+ |
| Ethyl methanesulphonate | 1 | A | + | + | + | + | 0 | |
| | 2 | W | + | 0 | 0 | + | 0 | |
| | 5 | W | 0 | 0 | 0 | 0 | 0 | 46+, 76+ |
| Hexamethylphosphoramide | 1 | A | + | — | — | + | 0 | |
| | 6 | A | — | — | — | — | 0 | |
| Hydrazine | 1 | A | + | — | + | + | 0 | |
| | 2 | W | 0 | 0 | 0 | 0 | 0 | 3+ |
| | 7 | P (L) | + | — | 0 | 0 | — | 46+ |
| MNNG | 1 | A | + | + | + | + | 0 | |
| | 2 | W | + | 0 | 0 | + | 0 | |
| | 5 | W | 0 | 0 | 0 | 0 | 0 | 46+, 76+ |
| Mitomycin C | 1 | A | + | + | + | + | 0 | |
| | 2 | W | — | 0 | — | — | — | |
| 2-Naphthylamine | 1 | A | + | — | — | — | 0 | |
| | 2 | A | + | + | 0 | + | 0 | |
| β-Propiolactone | 1 | A | — | + | — | + | 0 | |
| | 2 | W | + | 0 | 0 | + | 0 | |
| | 5 | W | 0 | 0 | 0 | 0 | 0 | 46+, 76+ |
| Safrole | 1 | A | + | — | + | + | 0 | |
| | 2 | A | — | 0 | — | — | — | |
| | 8 | H | 0 | 0 | 0 | 0 | 0 | 3+ 2+ |
| Urethane | 1 | A | — | — | — | + | 0 | |
| | 2 | A | — | 0 | — | — | — | |
| Vinyl chloride | 1 | A | — | — | — | — | 0 | |
| | 2 | P—(G) | + | 0 | 0 | + | 0 | |

Key for Table II.3

Lab.

1 Present study.
2 McCann *et al.* (1975) and references quoted therein.
3 Commoner *et al.* (1974).
4 Bartsch *et al.* (1976).
5 Brusick and Zeiger (1972).
6 McGregor (personal communication).
7 Herbold and Buselmaier (1976).
8 Green (1974).

Induction

A Aroclor induction of liver enzymes and S9 mix added.
W No S9 mix.
D 4-Dimethylaminoazobenzene induction of liver enzymes.
P Phenobarbital induction of liver enzymes and S9 mix added.
P— Phenobarbital induction but also activity without addition of S9 mix.
H Induction method unknown.
G Tested as a gas.
L Tested in liquid culture.
U Tested as urinary metabolites.

Key for Table II.3.—*continued.*

Body of table

| | |
|---|---|
| 0 | Not tested on that strain either in this laboratory or in other laboratories as far as can be determined. |
| + | + result. |
| − | − result. |
| 3+ | + on Strain TA 1530. |
| 2+ | + on Strain TA 1532. |
| 46+ | + on Strain G46. |
| 76+ | + on Strain C 3076. |

of carcinogenicity or non-carcinogenicity from the test result and from the classification (see Table 1 of main section) are given in the last 2 columns.

### DISCUSSION

The overall accuracy of the test in predicting the carcinogenicity of the compounds studied was high (91% for carcinogens and 94% for non-carcinogens. This supports the claims of Ames *et al.* (1975) that 85% (135/158) and McCann *et al.* (1975) that 90% (157/175) of the compounds tested were correctly predicted as carcinogens, and that in general the Salmonella mutation system is useful in predicting the carcinogenicity of organic chemicals.

This test has been able to distinguish between a number of structurally related carcinogen and non-carcinogen pairs of compounds (Purchase *et al.*, 1976 and Fig. 3 and 4 of main section).

About 50 compounds in this study have previously been tested by McCann *et al.* (1975). In general there is good agreement between the 2 studies. There are, however, some differences in the results in this study and those in the literature. These are highlighted in Table II.3. There are differences in detail between the techniques used by others and those in the present study, where no attempt was made to optimize amounts of S9 mix for a particular compound. The amount used was always constant (0·15 ml of S9 mix containing S9 fraction and cofactor in a ratio 27:63) whereas McCann *et al.* (1975) often optimized conditions for a compound. This validation assay was not conducted without microsomes whereas some other studies were. It is known (Ames *et al.*, 1975) that the mutagenic effect observed in this assay can be altered by variations in the techniques of enzyme induction and microsomal preparations, and these technical differences may well account for differences in results shown in the table.

From Table II.3 it can be seen that compounds may not be specific in the type of mutation that they cause, *i.e.* they are not specifically base-pair or frameshift mutagens. Examples from the table are 2-aminoanthracene, 4-aminobiphenyl, 6-aminochrysene and 2-naphthylamine. In the present study, 4-dimethylaminoazobenzene gave a positive result in all 4 strains. This is in contrast to the findings of Commoner *et al.* (1974) who were unable to obtain a positive response for this compound using the plate incorporation assay. They were, however, able to detect mutagenic activity in strain TA 1538 with the glucuronides from urine of 4-dimethylaminoazobenzene-fed rats after the urine was treated with β-glucuronidase and Taka diastase.

There was a negative result in the present study with dimethylnitrosamine, in agreement with the findings of Bartsch *et al.* (1976). On subsequent testing, positive results have, on occasions, been in agreement with the findings of Mattern (personal communication). Similarly, diethylnitrosamine was found positive in the present study but subsequently also found to be negative. Since it has been shown that the mutagenic activity of dimethyl and diethylnitrosamine can be detected reliably in a liquid culture assay (Bartsch *et al.*, 1976), the variations in test result with a plate incorporation test may be due to reaction of the active metabolites with agar, rather than DNA, to varying degrees.

Direct-acting mutagens such as ethyl methanesulphonate and MNNG are known to cause base-pair substitutions, but have been detected in all 4 strains with S9 mix in the present study. MNNG has been reported active in strains TA 1535 and TA 100 without S9 mix by McCann *et al.* (1975) but Ames *et al.* (1975) reported slight activity in strains TA 1538 and TA 98. Brusick and Zeiger (1972) reported a positive response with both methanesulphonate and MNNG with the base-pair-substituting strain G46 and also slight positive responses with the frameshift strain C3076.

Testing problems with hexamethylphosphoramide have been reported by Ashby *et al.* (1977).

Mitomycin C was tested under carefully controlled conditions (protected from light, freshly dissolved in ice-cold water) and gave a positive result. This was the only compound tested with prior knowledge of its identity.

Safrole has been reported positive in strains TA 1530 and TA 1532 (Green, 1974) in 3 strains in the present study, and strains TA 1950 and TA 1952 in a host-mediated assay after induction of enzymes with BHT (Green, 1974).

Vinyl chloride, dissolved in DMSO was found negative in this study, and by Rannug *et al.* (1974), whereas when it was tested as a gas we confirmed the positive result obtained by Bartsch *et al.* (1975) and Rannug *et al.* (1974).

The mean numbers of revertant colonies for the negative-control plates used in this study are comparable to those reported by Ames *et al.* (1975) for strains TA 1535 (20) TA 1538, TA 98 (40) but whereas Ames *et al.* (1975) observed about 160 colonies per plate with Strain TA 100 and McCann *et al.* (1975) reported 140 colonies per plate, 60 were seen in this study, and lower numbers have also been reported by Coombs *et al.* (1976). The strain of TA 100 used in this study was, nevertheless, ampicillin-resistant.

No attempt has been made to rank chemicals in order of carcinogenic potency on the basis of mutagenic activity.

### REFERENCES

Ames, B. N., Durston, W. E., Yamasaki, E. & Lee, F. D. (1973) Carcinogens or Mutagens: a Simple Test System Combining Liver Homogenates for Activation and Bacteria for Detection. *Proc. natn. Acad. Sci. U.S.A.* **70**, 782.

Ames, B. N., McCann, J. & Yamasaki, E. (1975) Methods for Detecting Carcinogens and Mutagens with the *Salmonella*/Mammalian Microsome Mutagenicity Test. *Mutation Res.*, **31**, 347.

Ashby, J., Styles, J. A. & Anderson, D. (1977) Selection of an *in vitro* Carcinogenicity Test for Use with Derivatives of the Carcinogen Hexamethylphosphoramide. *Br. J. Cancer*, **36**, 564.

Bartsch, H., Camus, A. & Malaveille, C. (1976) Comparative Mutagenicity of N-nitrosamines in a Semi-solid and in a Liquid Incubation System in the Presence of Rat or Human Tissue Fractions. *Mutation Res.*, **37**, 149.

Bartsch, H., Malaveille, C. & Montesano, R. (1975) Human, Rat and Mouse Liver Mediated Mutagenicity of Vinyl Chloride in *S. typhimurium* Strains. *Int. J. Cancer*, **15**, 429.

Brusick, D. J. & Zeiger, E. (1972) A Comparison of Chemically Induced Reversion Patterns of *Salmonella typhimurium* and *Saccharomyces cerevisiae* Mutants using *in vitro* Plate Tests. *Mutation Res.*, **14**, 271.

Commoner, B., Vithayathil, A. J. & Henry, J. I. (1974) Detection of Metabolic Carcinogen Intermediates in Urine of Carcinogen Fed Rats by Means of Bacterial Mutagenesis. *Nature*, **249**, 850.

Coombs, M. M., Dixon, C. & Kissonerghis, A. M. (1976) Evaluation of the Mutagenicity of Compounds of Known Carcinogenicity Belonging to the Benz(a)anthracene, Chrysene and Cyclopenta-(a)phenanthrene Series, using Ames's Test. *Cancer Res.*, **36**, 4525.

Garner, R. C., Miller, E. C. & Miller, J. A. (1972) Liver Microsomal Metabolism of Aflatoxin B1 to a Reactive Derivative Toxic to *Salmonella typhimurium* TA 1530. *Cancer Res.*, **32**, 2058.

Green, N. R. (1974) Screening of Safrole, Eugenol, their Ninhydrin Positive Metabolites and Selected Secondary Amines for Potential Mutagenicity. *Ph.D. thesis, Univ. Tennessee.*

Herbold, B. & Buselmaier, W. (1976) Induction of Point Mutations by Different Chemical Mechanisms in the Liver Microsomal Assay. *Mutation Res.*, **40**, 73.

McCann, J., Choi, E., Yamasaki, E. & Ames, B. N. (1975) Detection of Carcinogens as Mutagens in the *Salmonella*/Microsome Test: Part I, Assay of 300 Chemicals. *Proc. nat. Acad. Sci. U.S.A.*, **72**, 5135.

Purchase, I. F. H., Longstaff, E., Ashby, J., Styles, J. A., Anderson, D., Lefevre, P. A. & Westwood, F. R. (1976) Evaluation of Six Short-term Tests for Detecting Organic Chemical Carcinogens and Recommendations for their Use. *Nature*, **264**, 624.

Rannug, U., Johansson, A., Ramel, C. & Wachmeister, C. A. (1974) The Mutagenicity of Vinyl Chloride after Metabolic Activation. *Ambio*, **3**, 194.